# The impact of weight loss among seniors on Medicare spending

**DOI:** 10.1186/2191-1991-3-7

**Published:** 2013-03-20

**Authors:** Kenneth E Thorpe, Zhou Yang, Kathleen M Long, W Timothy Garvey

**Affiliations:** 1 Department of Health Policy and Management, Rollins School of Public Health, Emory University, Atlanta, GA 30322, USA; 2 Long Associates, Keswick, VA 22947, USA; 3 Department of Nutrition Sciences, Birmingham VA Medical Center, UAB Diabetes Research Center, University of Alabama at Birmingham, Birmingham, AL, 35294-3360, USA

## Abstract

**Objective:**

To examine the impact of temporary and permanent weight loss of 10% and 15% on 10-year and lifetime Medicare spending among adults with overweight and obesity aged 65 years and older. Weight loss of this magnitude is consistent with next generation anti-obesity medications recently approved by the Food and Drug Administration.

**Methods:**

We follow the approach of a longitudinal dynamic aging process model developed by our research team. This model considers the dynamic relationships between weight, chronic disease, acute medical events, functional status, mortality, health care utilization and spending among Medicare beneficiaries from age 65 until death. Using this model, we estimate baseline Medicare spending over the next decade and then over the lifetime of seniors with a body mass index (BMI) ≥ 27 with at least one weight-related comorbidity (overweight), and seniors with obesity having a BMI ≥ 30 and ≥ 35. We then estimate Medicare spending for this population between ages 65 and 70 over the course of a year, assuming 10% and 15% weight loss under alternative scenarios: with and without weight regain. (Weight regain is assumed to be 90% over a 10-year period.) The difference in spending between baseline (no weight-loss intervention) and the alternative scenarios represent potential gross savings to the Medicare program.

**Results:**

Permanent weight loss of 10 to 15% will yield $9,445 to $15,987 in gross per capita savings throughout their lifetime, and $8,070 to $13,474 over ten years. Similarly, initial weight loss of 10 to 15% followed by 90% weight regain will result in gross per capita savings of $7,556 to $11,109 over their lifetime, and $6,456 to $8,911 over ten years. Targeting weight loss medications to adults with obesity (BMI ≥ 30) produces greater savings to the Medicare program.

**Conclusion:**

Medicare can realize significant cost savings through anti-obesity medications that produce substantial weight loss, and as a result, reduce the progression to type 2 diabetes, and improve blood pressure and glycemic indicators in hypertensive and diabetic patients, respectively. Medications are currently excluded from coverage in the Medicare program, however, in light of potential savings and health benefits, may warrant consideration.

## Background

Obesity has been linked to a wide array of chronic health care conditions, including diabetes, hypertension, hyperlipidemia, cancer, heart disease, and musculoskeletal and pulmonary disorders, among others. The higher prevalence of chronic disease results in higher health care spending. In any given year, adults with obesity spend approximately 40% more on health care than normal weight adults [1,2], and people with diabetes spend 2.3 times more than people without diabetes [3]. Lifetime spending is also higher among overweight adults. Two recent studies have estimated that lifetime Medicare spending for adults starting at age 65 are 15–35% higher among adults with obesity as compared to normal weight adults [4,5]. There is substantial evidence supporting the beneficial effects of weight loss with respect to both obesity-related comorbid risk factors and mortality, which is why the National Institutes of Health (NIH) has established a weight loss target of 10% body weight from baseline within six months [6]. Unfortunately, there have been relatively few effective, long-term treatment options to choose from, as evidenced by the significant paucity of available treatments to fill the gap between lifestyle modification and bariatric surgery. Recently, new medications with better efficacy profiles have been FDA-approved for chronic weight management; however, reimbursement policies that are founded on a legacy of safety and efficacy concerns with previous anti-obesity medications are currently an impediment to access. Our hypothesis is that interventions designed to reduce the incidence and prevalence of overweight and obesity, or lower weight among adults with obesity, could both improve health outcomes and lower health care spending.

This study is the first to project the potential impact that a new generation of anti-obesity medications may have on both short term (10-year) and lifetime spending among the Medicare population (adults aged 65 and older) who are either overweight with at least one weight-related comorbidity (such as hypertension or diabetes) or are obese. Estimated savings are based on 10% and 15% weight loss, as recent clinical trials have shown these results to be attainable based on Intent-to Treat (ITT) and Completers analyses, respectively. We estimate these savings under five scenarios: baseline scenario without weight loss intervention; 10% and 15% initial weight loss followed by 90% weight regain over a 10-year period (i.e., “temporary” weight loss); and 10% and 15% permanent weight loss. The estimated savings assume Medicare would add weight loss drugs as a covered benefit; they currently are not. Estimated gross per capita savings do not include the costs of medications to the Medicare program, however, the impact of anticipated drug costs are included in the Discussion section.

The primary health benefits associated with weight loss include the long-term risk reduction for acute medical events as well as a reduction in the incidence of chronic disease. Hence, we estimated savings over the beneficiaries’ lifetime and over 10 years, as only evaluating immediate cost benefits would *under* estimate the value of weight loss intervention.

## Methods

The analysis presented below follows the approach of a Dynamic Aging Process (DAP) model developed by Yang [5]. This model profiles and approximates the dynamic aging process that involves the development of chronic diseases, deterioration of health status, fluctuation of body mass index, and Medicare expenditures for each beneficiary from age 65 to death. Using this approach, Yang and Hall demonstrate that lifetime Medicare spending among normal weight adults is about 17% lower than adults with obesity [5]. This model produces lifetime costs of obesity that are approximately 50% lower than a previous estimation from the Rand Future Expenditure (FEM) model using the same data source [4]; thus, we view the results of cost savings presented below as conservative.

To understand the lifetime Medicare savings from one year of baseline weight loss, we assume two scenarios that could bound the potential savings. First we assume initial weight loss of 10% and 15%, and based on the 10 year follow up of the diabetes prevention program (DPP), we assume 90% of the weight is regained over 10 years [7]. These scenarios produce the most conservative cost-savings estimates because weight regain in the DPP was both expected and observed with lifestyle modification. The one- and two-year clinical trials for a recently approved anti-obesity drug (phentermine/topiramate ER) provide no rationale for weight regain with on-going use of the medication, however, estimating some degree of weight regain provides a lower bound for cost savings. The second estimate assumes a sustained reduction in weight, which is consistent with 2-year published data demonstrating sustained weight loss with treatment adherence [8]. Recent evidence suggests physiologic mediators are responsible for the weight re-gain that often follows weight loss induced by lifestyle modification [9]. Therefore, much the way anti-hypertensive agents are maintained after a patient reaches a target blood pressure, or lipid-lowering drugs are maintained after reaching a cholesterol target, it is anticipated that a patient will need to continue on anti-obesity treatment indefinitely to maintain weight loss.

Using the DAP model described above, and following the previous publication by Yang and Hall, we first used Medicare Current Beneficiary Survey (MCBS) data from 1992 to 2001 to obtain the average 10-year and lifetime per capita Medicare expenditures as the baseline scenarios *without* any weight control intervention. We present estimates for 2012 to 2021 using the Congressional Budget Office projections of per capita Medicare spending [2]. Next, we conducted counter factual simulation under alternative scenarios, assuming those with BMI ≥ 27 and one weight-related comorbidity at age 65 lose 10% or 15% of their body weight, and calculated the average 10-year and lifetime per capita expenditures under the two scenarios (with and without weight rebound). Taking the differences between the 10-year and lifetime expenditures for the baseline and alternative scenarios, we obtained the average gross per capita savings for both temporary and permanent 10% and 15% weight loss. We repeated the analysis for a cohort of adults with class I (BMI ≥ 30) and class II (BMI ≥ 35) obesity.

## Results

Table 1 illustrates the differences in Medicare savings under each of the scenarios relative to baseline (no weight loss intervention). The first two columns depict savings based on temporary weight loss (10% and 15%) followed by 90% weight regain, and the last two illustrate the savings associated with permanent weight loss (10% and 15%). (The 2.4 million, 5.5 million and 3.3 million in each of the respective rows reflects the number of Medicare beneficiaries represented by each BMI category.)

**Table 1:** Lifetime and 10-year gross per capita Medicare savings from temporary and permanent weight loss among one cohort age 65–70

**Baseline BMI at age 65**	**10% Weight loss with weight regain (“Temporary”)**	**15% weight loss with weight regain (“Temporary”)**	**10% permanent weight loss**	**15% permanent weight loss**
BMI ≥ 27 + co-morbidity (2.4 million*)				
Lifetime	$7,556	$9,933	$9,445	$12,912
10 Years	$6,456	$7,831	$8,070	$10,180
BMI ≥ 30 (5.5 million*)				
Lifetime	$9,112	$10,304	$12,392	$14,116
10 Years	$7,446	$8,911	$9,053	$12,208
BMI ≥ 35 (3.3 million*)				
Lifetime	$7,799	$11,109	$13,496	$15,987
10 Years	$7,654	$8,534	$10,126	$13,474

*These numbers reflect the number of Medicare beneficiaries within each BMI category, which can be used to determine the available pool of aggregate savings.

These results demonstrate that permanent weight loss of 10–15% among adults with class I obesity (BMI ≥ 30) results in gross per capita lifetime Medicare savings of $12,392–$14,116 or $9,053–$12,208 over a 10-year period. Weight loss of that magnitude followed by 90% weight regain yields gross per capita lifetime Medicare savings of $9,112–$10,304 or $7,446–$8,911 over a decade.

For adults with class II obesity (BMI ≥ 35), we found 10–15% permanent weight loss could lead to even greater gross per capita lifetime Medicare savings of $13,496 to $15,987 or $10,126–$13,474 over 10 years. Weight loss of that magnitude followed by weight regain yields gross per capita lifetime Medicare savings of $7,799–$11,109 or $7,654–$8,534 over 10 years.

Finally, permanent weight loss of 10 to 15% for overweight beneficiaries (BMI ≥ 27) with diabetes or hypertension would be associated with a $9,445–$12,912 reduction in gross per capita lifetime Medicare spending or $8,070–$10,180 over 10 years. If the weight loss were temporary, i.e., it were followed by 90% weight regain, gross per capita lifetime Medicare savings would be $7,556–$9,933 or $6,456–$7,831 over 10 years.

Aggregate savings are dependent upon many factors, including the percentage of the Medicare population that takes the drug, adherence rates, weight loss results, etc. It is suggested that the reader develop his/her own assumptions and apply them to the total pool of available savings, which can be determined by multiplying the number of potential beneficiaries in each respective BMI category and the per capita savings. For example, from Table 1 it is evident that for class I obesity (BMI ≥ 30), the available pool of savings associated with 15% temporary weight loss (i.e., 90% of the weight is re-gained over 10 years), is $49 billion over a 10-year period. If that weight loss were permanent, the available savings pool is over $67 billion. Performing the same calculations using 10% temporary and permanent weight loss over 10 years yields a pool of available savings of almost $41 billion and $50 billion, respectively. For class II obesity (BMI ≥ 35), the available pool of savings for 15% temporary and permanent weight loss over 10 years is $28 billion and more than $44 billion, respectively; and for 10% temporary or permanent weight loss, the available pool of savings is $25 billion and over $33 billion, respectively.

## Discussion

These analyses estimate 10-year and lifetime gross per capita savings when Medicare beneficiaries with overweight (and at least one weight-related comorbidity) and obesity lose 10% or 15% of body weight, whether that weight loss is temporary or permanent. Over ten years, gross per capita savings to the Medicare program range from $6,456 to $13,474 depending on BMI category, percent weight loss, and sustainability of weight loss. Aggregate savings to the Medicare program will depend on the number of seniors with overweight and obesity who take the medication, and the cost of drug to Medicare each year. Sustained weight loss is difficult to achieve via lifestyle intervention due to physiologic mediators of appetite that encourage weight re-gain. Until recently, the lack of effective medical therapies has left only bariatric surgery as an option for some patients. However, new obesity medications have the potential to produce and/or sustain these levels of weight loss, and this provides rationale and relevance for our analyses. Two obesity drugs have recently been approved by the FDA, namely phentermine/topiramate ER (Qsymia®) and lorcaserin (Belviq®). The manufacturer of a third drug, bupropion SR/naltrexone SR (Contrave®), has reached an agreement with the FDA to conduct a pre-approval cardiovascular outcomes trial.

In Phase III clinical trials, all three drugs have been studied in patients with obesity (BMI ≥ 30 kg/m^2^) and overweight (BMI of 27–29.9) with weight-related co-morbidities, all of whom were provided lifestyle counseling and randomized to drug or placebo. While all three drugs produce significant weight loss relative to placebo, there is some variability in the degree of weight loss achieved. Specifically, 1-year studies demonstrate that lorcaserin treatment was associated with a 5.8% weight loss compared to 2.2–2.8% for placebo [10,11], and bupropion SR/naltrexone SR led to 6.1 to 6.4% weight loss as compared with 1.2–1.3% for placebo [12,13]. However, phentermine/topiramate ER produced the magnitude of weight loss that is in keeping with the savings upon which our model is predicated (i.e., 9.8% weight loss in patients with two or more comorbidities vs 1.2% for placebo and 10.9% in severe/extreme obesity vs 1.6% for placebo) [14,15]. Further, a long-term (2-year) clinical trial found sustained weight loss with continued treatment (SEQUEL), thus the gross per capita savings may be more in line with the permanent weight loss figures [8]. This trial also found reductions in fasting glucose and fasting insulin, and resulted in a 54% and 76% (depending on the dose) reduction in the annualized incidence rate for the progression to type 2 diabetes among all patients studied [8].

Because the fastest growing obesity category in the United States includes those individuals with BMI > 50, it is important to consider the potential efficacy of medication in this population. The EQUIP study is the only phase III trial that did not specifically exclude subjects with extreme obesity (i.e., BMI ≥ 45). The average BMI of the study was 42, which represents the prototypical patient who is referred for bariatric surgery. In this population, one-year weight loss at the highest dose of phentermine/topiramate ER was 10.9% (vs 1.6% for placebo) for the Intent-to-Treat (ITT) analysis and 14.4% for Completers (vs 2.1% for placebo) [15]. Importantly from both a clinical and cost perspective, significant weight loss (8–10%) occurred within 3 months of treatment, suggesting that non-responders may be identified early. Two-thirds of treated subjects who completed one year of therapy (“Completers”) achieved the NIH target of ≥ 10% of body weight, and almost half lost ≥ 15%, regardless of baseline BMI, suggesting that individuals with extreme obesity can achieve significant weight loss without surgery.

The CONQUER trial evaluated patients with a minimum of two obesity-related co-morbidities [14]. This is somewhat analogous to the Medicare population, as 93% of Medicare beneficiaries have at least one obesity-related condition [16]. The highest dose of phentermine/topiramate ER achieved 9.8% and 12.4% weight loss after one year in the ITT and Completer analyses, respectively, (vs 1.2% and 1.6% for placebo), and showed comparable weight loss regardless of age (~10% of subjects were ≥ 65 years of age) [14]. The one-year extension of this study (SEQUEL) demonstrated the durability of the response with sustained weight loss of 10.5% (vs 1.8% for placebo) for the ITT analysis over the two-year period [8]. In each of these studies, weight loss of this magnitude was associated with improvements in cardiometabolic risk factors, including blood pressure, fasting glucose, fasting insulin and lipid profiles, which is in keeping with the risk reductions in our model upon which the Medicare savings are based.

Table 1 indicates that each senior with obesity who permanently loses 15% of weight saves Medicare, on average, more than $12,000 in gross savings over a 10-year period. It is therefore evident that effectively treating obesity with medication can be cost-effective. Taking the average of the published Wholesale Acquisition Costs (WAC) for both the recommended and top dosage strengths of phentermine/topiramate ER, and assuming a Medicare Part D tier-3 co-pay of $70 per month, *net* per capita savings to Medicare are anticipated to be almost $3,000 over 10 years. Similar calculations for beneficiaries with class II obesity (BMI ≥ 35) show even greater *net* per capita savings of almost $4,000 over 10 years. These net savings assume continual treatment with phentermine/topiramate ER throughout the 10-year period because it is now recognized that obesity is a chronic disease that requires ongoing intervention to sustain weight loss. The chronic nature of obesity treatment is recognized in the approved indication for phentermine/topiramate ER, which is an adjunct to a reduced calorie diet and increased physical activity for *chronic* weight management in adults with obesity (BMI ≥ 30), or overweight (BMI ≥ 27) with at least one weight-related comorbidity. The objective of pharmacotherapy is to counteract the underlying pathology of obesity, which is the dysregulation of physiologic mediators that affect appetite and satiety. Discontinuation of pharmacotherapy after successful weight loss removes this physiologic counterbalance and is likely to lead to weight regain; hence, there is no maximum time with respect to treatment duration, regardless of the resulting BMI. This is analogous to the continuation of anti-diabetic or anti-hypertensive therapy after reaching an HbA1c target or adequately controlling blood pressure. In both situations, therapy would be continued as the underlying pathology is still present.

It is important to note that these savings do not take into consideration the reduction in dose or number of concomitant medications used to treat the complications of obesity, such as anti-hypertensive agents or drugs used to treat type 2 diabetes. Clinical trials demonstrated that patients taking phentermine/topiramate ER achieved reductions in HbA1c and decreased blood pressure, despite reductions in related medications aimed at reaching recommended therapeutic targets.

## Conclusion

The Affordable Care Act added some additional preventive benefits into the Medicare program. These benefits include the elimination of cost sharing for certain clinical prevention benefits, a new health risk appraisal, and a personalized care plan. One specific benefit is coverage of intensive behavioral therapy (IBT), which reimburses primary care physicians for IBT of beneficiaries with obesity for up to one year, provided the patients lose at least 6.6 pounds during the first six months (approximately 3% of body weight using average baseline weight of clinical trials). Yet, the current Medicare wellness benefit remains incomplete since it does not include coverage for weight loss medications that have demonstrated the ability to significantly improve risk factors associated with cardiometabolic disease. Now that Medicare reimburses primary care physicians for IBT, it would seem logical to also cover effective anti-obesity agents since the combination of the two therapies is more successful than either one individually. This combination could prove a valuable strategy for providing sustained weight loss, and with it, a reduction in overall health care expenditures.

Medicare also provides coverage for bariatric surgical procedures for patients with a BMI ≥ 35 who have at least one weight-related comorbidity. New clinical developments include the approval of medications that can produce weight loss of almost 15% in this population of patients with severe and extreme obesity. Including these medications in the Medicare benefit package would fill the large treatment gap that exists between IBT and bariatric surgery.

There are caveats with our results. The first is the relationship of drug dosage, treatment adherence and the duration of weight loss. While weight loss produced by phentermine/topiramate ER has demonstrated durability over a two-year period of treatment, the results of longer-term therapy are unknown, and the estimated savings would be lower if weight loss is not sustained. The top dose led to greater weight loss results, particularly in severe obesity, and patients who adhere to a treatment regimen are more likely to achieve 10% and 15% weight loss, and sustain it over time. Therefore, we believe our two scenarios, one with an initial weight loss followed by 90% weight regain over 10 years, and the other with permanent weight loss provide a reasonable bound of the potential savings. In addition, aggregate savings will depend on how many Medicare beneficiaries in a particular group (e.g. BMI ≥ 30) use the weight loss drug and achieve the same average result. FDA labeling for newly approved agents requires a minimum 5% weight loss within a designated period of time or the patient should be considered a non-responder and the drug discontinued. This should guard against excessive medication costs without the commensurate health benefits. Finally, although Table 1 figures do not include the cost of the drug to the Medicare program and beneficiaries, published WAC pricing and assumptions with respect to average monthly co-pays provide reasonable guidance for determining cost-effectiveness when compared to per capita cost savings.

It is clear that 10–15% weight loss among individuals with obesity has the potential to produce significant savings for the Medicare program. Currently, anti-obesity drugs are an excluded category for coverage, while IBT and bariatric surgery are covered benefits. This has resulted in a significant treatment gap for the majority of patients with obesity. The results of our analyses confirm that greater savings in health care spending will be achieved by targeting the population with obesity as a priority for weight management. It appears that this could be achieved with the advent of effective anti-obesity medications recently approved or under review by the FDA.

## Abbreviations

DAP: (Dynamic aging process); BMI: (Body mass index); NIH: (National Institutes of Health); ITT: (Intent-to-treat); IBT: (Intensive behavioral therapy).

## Competing interests

Dr. Thorpe is a consultant to VIVUS, Inc. Dr. Garvey is a consultant to VIVUS, Inc. Ms Long is a consultant to VIVUS, Inc. and owns a small amount of stock in a retirement account. Dr. Yang has no competing interests.

## Authors’ contributions

KT conceived and designed the research study, supervised the research team and provided initial draft of the manuscript. ZY participated in the design of the study, conducted the research, acquired the data and provided analysis. TG provided clinical interpretation of the data and results, and edited the manuscript from a clinical perspective. KL provided policy interpretation of the data and results and critically edited the manuscript from both a policy and clinical perspective. All authors have read and approved the final manuscript.

## Authors’ information

KT is a public health policy expert and is widely published in that arena. He is the Former Deputy Assistant Secretary for the U.S. Department of Health and Human Services (HHS), and currently is Chair of the Department of Health Policy and Management at the Rollins School of Public Health, Emory University. He is also the Chairman of the Board for The Partnership to Fight Chronic Disease (PFCD), which is a coalition of hundreds of patient, provider, community, business and labor groups, and health policy experts, all committed to raising awareness of chronic disease—the number one cause of death, disability and rising health care costs. One important goal is to educate policymakers so that they will implement comprehensive healthcare reform that addresses chronic disease from a prevention standpoint, which is more cost-effective than treating chronic disease after it has occurred, saves more lives and also improves quality of life.

TG is a widely published, internationally recognized expert in the fields of metabolic, molecular, and genetic pathogenesis of insulin resistance, type 2 diabetes and obesity. He was a member of national research review committees for the Juvenile Diabetes Research Foundation, the American Diabetes Association, the VA Merit Review Program, and the National Institutes of Health. He was a standing member of the Metabolism Study Section at NIH from 1998-2002, and has chaired several ad hoc NIH study sections. TG currently serves on the editorial boards of Diabetes, and has previously served in this capacity for the Journal of Clinical Endocrinology and Metabolism and Diabetes Reviews. He is a member of the American Society for Clinical Investigation, the Association of American Physicians, the Endocrine Society, and the American Diabetes Association, and the North American Association for the Study of Obesity.
